# The perceived benefits and effectiveness of patient feedback systems in strengthening patient-provider relationships in Rural Tanzania

**DOI:** 10.1186/s12913-023-10198-z

**Published:** 2023-11-03

**Authors:** Kahabi Isangula, Eunice S. Pallangyo, Eunice Ndirangu-Mugo

**Affiliations:** 1 School of Nursing and Midwifery, The Aga Khan University, Dar Es Salaam, Tanzania; 2 School of Nursing and Midwifery, The Aga Khan University, Nairobi, Kenya

**Keywords:** Patient feedback mechanisms, Complaints mechanisms, Healthcare, Patient-provider relationships, Trust, Rural, Tanzania

## Abstract

**Introduction:**

Patient feedback system (PFS) forms an important entry point for the medical personnel and healthcare administrators to identify healthcare service delivery gaps and develop responsive interventions. This may foster patient trust consequently increasing healthcare-seeking, engagement in decision, continuity, and satisfaction. However, research on the PFS in rural primary healthcare settings appears limited.

**Objective:**

The paper examines the perceived role and effectiveness of PFS in improving therapeutic relationships building on the recent research on patient-provider relationships in rural Tanzania.

**Methods:**

The paper examines the findings of qualitative descriptive research conducted in the Shinyanga Region which employed a human-centred design (HCD) approach to co-create an intervention package for improving nurse-client relationships between January and September 2022. The study used semi-structured interviews in Swahili to first explore drivers of poor provider-patient relationships with purposefully selected providers, patients, and administrators. The findings guided the co-designing of an intervention package in subsequent HCD steps. Interviews were concurrently translated and transcribed, then systematically coded to facilitate the development of themes using a deductive thematic analysis approach.

**Results:**

PFS emerged as one of the key themes in the deductive analysis when examining factors shaping provider–client relationships. The PFS theme was characterized by three major subthemes, which included perceived benefits, availability and accessibility, and perceived effectiveness. The perceived benefits of PFS cited by most participants included: reducing patients’ confusion around the complaints process, promoting patients’ positivity towards providers and hospitals, and reducing tensions between patients and providers. Suggestion boxes (SBs) were the most frequently cited PFS, but there were widespread concerns and disagreements among participants about their accessibility and effectiveness. Despite the providers (nurses) and administrators describing SBs as widely available, they stated that they had not received feedback or complaints from patients for a very long time. In contrast, most patients stated that SBs were either unavailable or ineffective in many facilities, with concerns about non-user friendliness and lack of responsiveness as the main issues when discussing effectiveness.

**Conclusion:**

Despite the many benefits of PFS in improving healthcare service quality, their availability, user-friendliness, and responsiveness still pose challenges. A call is made to providers, health administrators and researchers to prioritize the PFS as both a useful entry point to reducing tensions in therapeutic relationships and, a tool for improving patient service uptake, continuity of care and satisfaction.

**Supplementary Information:**

The online version contains supplementary material available at 10.1186/s12913-023-10198-z.

## Background

A considerable amount of literature has been published on persistent patient dissatisfaction with providers’ care in Saharan Africa (SSA). For instance, the perceptions of provider’s technical incompetence (skills, reliability, assurance, confidentiality, patient engagement) and behavioural incompetence (demeanours, empathy, communication skills, i.e., language, respect) dominate in patients’ descriptions of dissatisfactions in Tanzania [1–5], Uganda [6] and Malawi [7, 8]. This trend appears common even in middle-income African countries e.g., South Africa [9–11]. Consequently, there has been increasing media coverage and political interventions towards providers’ technical and behavioural incompetencies in public healthcare facilities [12–15]. Political interventions often involve the suspension of providers accused of malpractices [13–15] and this has continued to contribute to the politicization of medicine and weaken the functionality of medical professional bodies [16, 17]. This may partly contribute to poor patient healthcare service uptake, engagement in decision making and continuity with care, particularly in diseases requiring ongoing therapeutic relationships [18–20] as well as reduced providers’ work morale, commitment, and job dissatisfaction with many desiring to quit [21–25]. The question of how to reduce patient dissatisfaction, particularly in low-income rural contexts remains largely unanswered.

The patient feedback system (PFS) presents a valuable but under-researched tool for addressing patient dissatisfaction with providers in primary healthcare (PHC) settings. PFS can be defined as a system that keeps track of clients’ or patients' opinions about the standard of care received with the aim of assisting hospitals and providers in learning how to enhance procedures and the patient experience [26]. The feedback received may include patient satisfaction levels, experiences, opinions, and evaluations of the accessibility, continuity, and quality of healthcare services received [26]. PFS continue to form an important tool for improving provider-patient relationships and the quality of care. A well-planned PFS is an important entry point for medical personnel and administrators to identify healthcare service delivery gaps and develop responsive interventions to address them [27, 28]. Effective PFS has been regarded as part and parcel of a good healthcare institution and gives the patients, as customers, an opportunity to monitor and report the quality of care, providers’ incompetence, dissatisfactions, and maltreatment [29–31]. In a competitive healthcare market, there is a need for providers and healthcare administrators to understand their patients' experiences of healthcare services and what they need. This may foster patient trust and loyalty within therapeutic relationships consequently increasing healthcare-seeking, and empowering engagement in decision, continuity, and satisfaction [28]. Effective PFS within healthcare settings, therefore, forms a critical entry point to addressing patient concerns in therapeutic relationships without needing political interventions.

Despite their value, studies on PFS in rural PHC settings appear limited. For instance, while other PFS such as exit surveys have been widely reported in the UK [27], only suggestion boxes (SB) have been examined from advocacy standpoints in Tanzania. One survey by Sikika, a health advocacy entity [32] documented fair availability of SBs in urban districts of Dar Es Salaam and widespread unavailability in rural districts of Dodoma (Mpwapwa and Kondoa) and Pwani (Kibaha) Regions. Concerns about the ineffectiveness of SBs in the few healthcare facilities where available were widespread among participants of this survey. Beyond the realm of advocacy, to date, there has been limited research documenting the impact PFS could have on patient-provider relationships. This paper aims to examine the potential role and effectiveness of the PFS in improving therapeutic relationships.

## Methods

### Design

The paper draws from the findings of a recent qualitative descriptive study on patient-provider relationships in rural Tanzania. The parent study employed a human-centred design (HCD) approach as an investigative framework with a mix of qualitative methods (focus group discussions (FGDs), key informant interviews (KIIs) and consultative meetings) to co-create an intervention package for improving nurse-patient relationships between January and September 2022 [33, 34]. Co-designing of the intervention package using HCD was guided by the following: (i) Overall research question: *What are the drivers of poor nurse-client relationships in maternal and child health (MCH) care in rural Tanzania?* and (ii) Design question: *What is the best intervention co-developed by nurses and clients for strengthening nurse-client relationships to address these drivers’ considering feasibility and acceptability?* A qualitative descriptive approach [35] was appropriate for this inquiry as it aimed to develop an understanding and describe nurse-patient relationships without testing an existing theory. This approach offered an effective way of gaining a deep and rich understanding of nurse and patient perceptions and experiences of drivers of poor relationships in their context, as this may differ from other contexts in terms of culture, expectations, and resources within healthcare settings.

### Settings

The study was conducted in Shinyanga, a region located in the Lake Zone in Tanzania. Isangula [36] offers a detailed description of the region. Briefly, Shinyanga falls within the low-income category of the regions in Tanzania. It is administratively divided into 6 districts: Shinyanga Municipal Council (MC), Shinyanga District Council (DC), Kishapu DC, Ushetu DC, Kahama MC, and Msalala DC. The rationale for choosing Shinyanga is twofold. First, ethnically, the region is predominantly inhabited by Sukuma, who share a range of sociocultural beliefs and practices with minimal diversity. Due to its near homogeneity, the region was a perfect exemplar of many other rural regions of Tanzania. Second, despite a number of capacity-building interventions, local data indicate enormous concerns about poor nurse-client relationships in healthcare settings [34, 36]. Within the Shinyanga region, Shinyanga MC was purposefully selected because patients in this district have greater access to both the formal health care system (mostly public and few private and faith-based facilities) and traditional care [36].

### Sampling and recruitment

The study involved 9 FGDs with purposefully selected nurses (30) and patients (36) and KIIs with selected healthcare administrators (12) in Shinyanga MC making a total of 78 participants [33, 34]. Purposive sampling was used because statistical representation was not the primary goal. During participant recruitment, no strict criteria were applied other than the inclusion of patients who were seeking maternal and child healthcare (MCH) at the time of the study (see [33]).

The process of recruitment of FGD participants began with careful consideration of ownership and level of healthcare facilities where participants receive MCH care. The Shinyanga MC medical officer was then visited to request permission to visit the facilities. The information about the study was then given to the healthcare facility managers during physical introduction visits. One provider with strong interpersonal communication skills was identified within the chosen institution with the help of the facility manager to act as an enrolment assistant. To aid in the recruitment of providers and MCH clients, each suggested enrolling assistant was briefed on the aims of the study and subsequently omitted from FGDs. The enrolling assistant informed participants who expressed interest in the study during clinical meetings (to recruit providers) and MCH visits (to recruit clients) and registered them. Thereafter, research assistants made additional visits to set up and conduct interviews. Recruitment of KII participants commenced with acquiring the phone numbers from the regional and district medical offices followed up by making the initial phone contact with MCH administrators. Administrators were provided with study information over the phone then interviews were scheduled with those who were willing to engage after considering their preferences for time and location. There were no refusals documented because participants were given adequate information, allowed to ask questions, and received adequate responses, and self-registered to participate in the study.

### Data collection tools

The original semi-structured FGDs and KII guides were developed and translated through a consultative process involving experts at Aga Khan University. The English versions of the interview guides were translated into Swahili language then back translated to English and checked for conceptual equivalence. The questions and prompts related to PFS were added to the original tool after a couple of interviews (Supplementary File 1) and they included the perceived benefits, availability and accessibility, patient usage, complaint handling, and feedback provision. Pre-testing was conducted in carefully selected settings to refine the interview guides, involving rephrasing questions and adding more probes, and ensuring their readiness for actual data collection.

To ensure the collection of rich data, three research assistants who are native Swahili speakers and fluent in English with a Diploma in medical and social sciences were recruited and trained on the use of interview guides and techniques pertaining to this study. Close and supportive supervision of research assistants and daily debriefings were conducted throughout the data collection and analysis stages to ensure data quality.

### Data collection

Upon arrival at the selected healthcare facilities, research assistants arranged and conducted interviews in a quiet, isolated room that was cut off from the normal clinics. Participants were given information about the study, potential risks, and benefits of participation prior to the start of FGDs and KIIs (an information sheet was included in the interview guide). Before the interview, verbal consent for voice recording was requested and recorded as part of the interview transcript. Then, the audio-taped interview sessions lasting for approximately 45–60 min were conducted excluding the time for the consent process. Additional interviews were deemed unnecessary because the study sample size was adequate to achieve data saturation. After the interviews, research assistants prepared field notes and shared them with the principal investigator (PI).

### Data management and analysis

Data transcription and back translation to English occurred simultaneously by the research assistants. After transcription and translation, the interview transcripts were cross-checked by the research team (three senior medical and public health experts) to ensure that participants’ worldview was not lost during translation. The interview transcripts were then de-identified, and pseudonyms were generated for each participant. The data were then uploaded into NVivo software by the research assistants for thematic coding. The deductive thematic analysis was then conducted by the research team and was based on the approach described by Braun and Clarke [37], and began after the first few interviews and continued as more data were gathered. More specifically, the PI deductively generated an initial list of codes from data extracts of the first three transcripts (deductive thematic coding). Then, these codes were reviewed by the research team who had independently reviewed selected transcripts generating a consensual list of codes. The PI continued coding the rest of the transcripts, refining, and generating more codes upon coming across a new segment of data that could not fit into the initial codes. Codes were then sorted into potential subthemes and themes, followed by collation of all relevant coded data extracts within identified themes. Throughout coding and refinement, the peer consultation was maintained to reflect on the subthemes and themes generated. PFS emerged as among the key themes during the coding of the first few interviews and additional information was gathered in subsequent interviews. Codes related to this theme were merged into several subthemes including their perceived benefits, availability, accessibility, and effectiveness when examining the factors shaping patient-provider relationships. These subthemes formed the basis for this paper as detailed in the results section.

## Findings

This paper examines the findings of 9 FGDs and 12KIIs whose accounts included issues pertaining to PFS. Among the participants 90% were Female, 50% were aged 31–40 years and the majority of clients (61%) had primary or no formal education (Table 1).

**Table 1:**
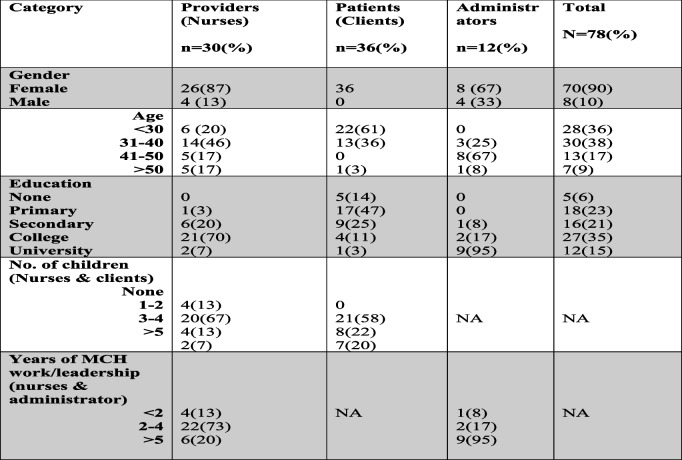
Participants’ demographics

### Overview of the findings

Participants discussed PFS from three standpoints: (i) the importance of feedback mechanisms in patient-provider relationships, (ii) their availability and accessibility and (iii) whether existing feedback mechanisms are effective (Table 2). Each of these is examined in detail below.

**Table 2:** Summary of themes and subthemes emerging from the analysis

THEMES	SUBTHEMES	REMARKS
**Contributors of poor nurse-client relationships**	• Nurse contributors • Patient contributors • Health system contributors	Published elsewhere [34]
**Patient Feedback Systems**	• Perceived benefits • Availability and accessibility • Perceived effectiveness	Forming the basis for this manuscript
**Suggestions for improving nurse-client relationships**	• Suggestions for providers • Suggestions for patients • Suggestions for health system and policy	Partly published elsewhere [34]

#### Subtheme 1: The perceived benefits of PFS in the therapeutic relationship

Looking across transcripts, the benefits of PFS were described as threefold. First, some patients describe PFS as necessary in the healthcare system because they have always faced unfavourable experiences and are mostly unaware of where to complain or report. For instance, one of the MCH clients (herein referred as to Regina, a farmer), described encountering favouritism at the MCH clinic which created dissatisfaction. Regina mentioned that she was unaware of where to complain/report the dissatisfaction. Regina commented:

“… *I went there [facility name], and I did not have someone I personally know to facilitate quick access to care. I ended up waiting for a very long time. But other patients come to the clinic accompanied by the provider whom they personally know...or a nurse comes with a patient they know each other and favours them by taking him/her to see a doctor without considering those who are queuing ... There may be only three patients remaining on a queue but a nurse comes with someone who she personally know and bypasses all of you even if it was your turn to see a doctor. They go to see a doctor and receive treatment before you although they found you there…Aah... I did not know where to complain or report... I just beared with it because I just wanted to get treatment.” (Patient 16, Farmer)*

Secondly, some participants went further to describe PFS as a useful tool for preventing the bad reputation of the healthcare providers and healthcare institutions and providers from reaching the community. A reference was made to instances when clients are dissatisfied and carry the dissatisfaction to their social networks consequently promoting the bad reputation of providers and healthcare facilities within communities which may negatively impact healthcare-seeking practices. For instance, nurses and administrators spoke about ineffective PFS as a reason for negative experiences to reach the community through patients’ social networks which may in turn affect healthcare service utilization:

*“[Ineffective feedback mechanisms] affect utilisation of health services...because you [provider] can answer a patient something which she may perceive as bad language and she goes tell other people in the community about your language. She will not keep quiet, and, when the patient tells other people, it may destroy the reputation of both the facility and the provider.”( Nurse 4).*

*“Poor system of gathering patient complaints may affect the uptake of healthcare services when patients go to the community and spread the negative experiences they encountered in the hospitals. The people who heard the story may decide not to seek care from the facility or provider who contributed to such bad experiences.” (MCH administrator 6)*

Finally, some participants described PFS as a way of preventing patients from utilizing other forums for airing their dissatisfactions that emerged from therapeutic encounters with providers within healthcare settings. This is particularly important as patients were said to frequently utilise the media and political meetings to air their negative experiences in healthcare settings because of ineffective PFS. An example was a clinical officer and a manager in a health centre who suggested that ineffective PFS can contribute to patients to ‘*complain everywhere, particularly to politicians’* which may further create tensions between patients and providers (MCH Administrator 11). In addition, a facility governance committee chairperson (HFGC) commented:

*Patients tend to complain to politicians because facilities do not have an effective system for collecting their worries and concerns. If the facilities improve the complaints mechanisms, then patients have no reason to go to politicians (MCH Administrator 2)*

Taken together, the participants’ accounts suggest that PFS may enhance both the provider and health facility’s reputation within the community and create an opportunity for providers to address patients’ dissatisfactions in PHC consequently impacting the patient-provider relationships positively.

In examining the findings related to the effectiveness of PFS, participants’ accounts are heuristically presented based on the Ombudsman [38] criteria: *availability, access and utilization, responsiveness,* and *user satisfaction*. Each of these is examined under two additional subthemes emerging from the analysis.

#### Subtheme 2: Availability, accessibility, and utilization of PFS

Regarding availability and accessibility, the participants of this study described suggestion boxes (SBs) as a prevalent form of PFS in Shinyanga. However, disagreements emerged between providers and healthcare administrators, and patients regarding the availability of the SBs the in public healthcare facilities. On the one hand, providers and healthcare administrators described SBs as both available and effective. For instance, one nurse affirmed that SBs are both available in “*every health facility*” and have a significant “*contribution to gathering [patient’s] opinions” (Nurse 22)*. However, upon more probing, the nurse acknowledged that only “*some [of] healthcare facilities have suggestion boxes”*. On the other hand, patients described SBs as mostly unavailable and ineffective where available. For instance, Mabula (pseudonym for Patient 31) considered SBs as both “*not there”* because they “*suddenly vanished at the [name] hospital”* and *“not the right approach”* to gather patient concerns. Some providers suggested the availability of other forms of PFS. For instance, a district healthcare manager (MCH administrator 12) described routine health education sessions in healthcare as another approach used to gather patients’ voices on “*whether [a patient] wasn’t satisfied with the services we offered or faced bad language or her/ expectations were not met”*. Similarly, an assistant medical officer (MCH administrator 9) described “*frequent meetings with community leaders”* as alternatives. However, the effectiveness of these alternative PFS was not established.

Furthermore, with reference to SBs, the disagreements on the availability of PFS were accompanied by disagreements on their utilization. Whilst providers and healthcare administrators described the availability of SBs, they all affirmed not having received patients’ suggestions or complaints so far. For instance, a medical doctor and a manager of one healthcare facility (MCH administrator 4) acknowledged that *“the mechanisms of offering suggestions are challenging because [they] have not received any suggestions”.* In support, Joyce (pseudonym for a clinician and a manager of another healthcare facility affirmed to “*never [have] received any complaints*”. Only one client (Patient 4, a farmer) describes having given *“suggestions on many occasions”.*

The reasons for the non-utilization of SBs by patients were described by participants as fivefold. The first reason for the non-utilization of SBs where available is the lack of awareness of their availability among patients. It is for this reason, one participant (Nurse 24) proposed advertising or putting a signboard at the healthcare facility to indicate their location. The second reason for the non-utilization of SBs where available is the absence of tools such as “*a pen and paper”* (MCH administrator 1) as patients do not carry these items when visiting the healthcare facilities for medical services. The third reason for the non-utilization of SBs where available is the patients’ avoidance of becoming the source of the providers’ punishment. There were fears that complaining about a provider would condemn him/her for punishment by supervisors. For instance, Pendo (pseudonym for Patient 10, a farmer) described uncertainties in offering suggestions in the future because *“it is not [her] behaviour to just give comments for health workers to be punished”.* This suggests that some patients may fear complaining because they do not want providers to be punished because of their complaints. Relatedly, the fourth reason for the non-utilization of SBs where available is patient fear of provider retaliations. Some patients suggested that when complaints are handled by the same people (detailed below), it may impact the quality of services they receive in future encounters. The fifth and final reason for the non-utilization of SBs, where available is patients’ uncertainty about who acts on the complaints. This is described next.

#### Subtheme 3: Effectiveness of PFS (Responsiveness and user satisfaction)

Responsiveness of PFS relates to when complaints are worked upon by responsible entities, desired changes are instituted, and feedback is given to the patients. Patients’ accounts indicated uncertainties on whether their complaints are taken into consideration. For instance, Neema (pseudonym for Patient 35, a businesswoman) suggested that *“some of the health facilities have suggestion boxes but the health workers forget about them…and…they are not being opened”*. Related to this, Yonge (pseudonym for Patient 8, a farmer) does not think the *“leaders are working on the suggestions”.* In support, a farmer who described offering suggestions/complaints on several occasions (Patient 4 above), was *“not sure if [complaints] were acted upon”* because she neither saw the changes she recommended being instituted nor received feedback from the providers. The farmer believed the complaints are ‘*handled by the same people who are at the centre of patients’ complaints’* which may contribute to not working on them. This may explain why some participants recommended the establishment of a separate entity or organization to gather, communicate and report on the complaints in the healthcare sector. One participant commented:

*The problem is that the suggestions we give go to the same people...it is not possible to address them. They may just burn them away. If the suggestions are about someone who is the same person reading them, s/he may just discard them and keeps quiet (Patient 2, Farmer).*

Upon further inquiry, some clients indicated not making a follow-up to see if changes are instituted. Regina (Patient 16, cited above) affirmed that she “*played [her] part by presenting what was disturbing [her], did not follow up to see implemented”.* This raises a question as to whether patients are to follow up on the implementation of their suggestions or whether providers should seek to provide patients with reports on the implementation of their feedback.

With the perceived ineffectiveness of SBs, some patients suggested an independent agency (as indicated above) or private and confidential one-on-one discussion as suitable alternatives. For instance, a housewife (Patient 18) refered to the interview for the current study as an ideal PFS. The absence of or ineffectiveness of PFS in the study settings may explain why participants utilised the interview as an opportunity to present many concerns related to both interpersonal and non-interpersonal aspects of care. One participant commented:

*The leaders can address these challenges by doing something like what you are doing...talk to patients. One of the things that influenced you to do this (talk to patients) is finding a way of improving health care services. You have been hearing that patients have lots of complaints...So, you can’t know about these things if you haven’t met patients and listened to their concerns. It should be done this way. The way you are doing this- talking to patient-one-on-one. (Patient 28, Businesswoman)*

Collectively, participants’ accounts suggest both unavailability in many facilities and ineffectiveness of existing PFS in few facilities where available. Also, all providers interviewed affirm having received no complaints so far and only one patient described having offered suggestions on some occasions. Patients’ awareness of the existence of PFS, uncertainties and fears, structural barriers, and non-responsiveness of PFS appear to further limit the effectiveness of existing SBs wherever available. These accounts suggest that patients may be dissatisfied and distrustful of the PFS approach which explains why some recommended the need for an independent entity to handle complaints and feedback.

## Discussion

This paper is based on a study that employed a human-centered design (HCD) approach to enhance nurse-client relationships, with a specific focus on investigating the factors influencing therapeutic relationships in MCH care [33, 34]. The overarching goal of the parent study was to collaboratively design an intervention package (prototype) aimed at improving nurse-client relationships within the rural Shinyanga region of Tanzania. This process involved a series of iterative HCD steps and engaged various key stakeholders, including nurses, clients, and MCH administrators. These stakeholders worked together to customize solutions for intricate issues that impact provider–client interactions in MCH care [34]. While the study identified PFS as a significant theme in the analysis, previous publications stemming from the research did not delve into a comprehensive examination of this aspect.

This paper used the accounts of some providers, patients, and administrators in formal care to offer a detailed analysis of the perceived benefits and effectiveness of the PFS in rural Tanzania. Consistent with some literature on the patient-provider relationships [6–8, 34, 36], the accounts of participants in the present study suggest that patients are facing unfavourable experiences with providers. Some of these unfavourable experiences involve favouritism in MCH clinics where longer waiting time appears to be common [6–8]. However, most patients describe being unaware of where to express their complaints, dissatisfactions, or feedback. Some patients and providers considered PFS as mostly non-existent, and some considered them ineffective in a few healthcare facilities where suggestion boxes are available. The absence and/or ineffectiveness of PFS may be fuelling patients to use other forums for expressing their dissatisfaction with care [12–17]. This may partly explain why patients appear to prefer using media outlets and political forums to express their dissatisfactions upon facing interpersonal challenges in healthcare settings [12–15, 36]. Furthermore, the use of media and political forums for expressing dissatisfaction in healthcare settings has the potential to fuel patient distrust in providers and government-owned health institutions and is likely to impact service uptake and continuity with care [5]. This suggests that there is a need for providers and administrators not only ensure the availability of diverse PFS but also effective systems for gathering patients’ feedback in PHC settings.

In places where PFS are cited as available, suggestion boxes appear to be prevalent as they were frequently referred to by most providers and healthcare administrators as compared to patients. This is consistent with the findings of a survey by Mahindi et al. [32] who reported suggestion boxes to be the most prevalent PFS in healthcare settings cited by 80% of the respondents. Similar to the findings presented, there is a tendency among medically trained participants to affirm the availability and effectiveness of suggestion boxes while non-medically trained participants affirmed unavailability and ineffectiveness [32]. Such disagreement raises two major questions. First, whether some providers are content with dysfunctional PFS within their institutions. The tendency of some providers to insist on PFS as available and effective contrary to patients suggests that some providers may be willing to conceal or turn a blind eye to the dysfunctions of PFS within their health facilities. This points to a suggestion that some providers may resist changes that intend to improve the effectiveness of PFS or when new PFS are introduced. This implies that efforts to improve the effectiveness of PFS need to include activities that advocate for healthcare providers to recognize their value in improving the quality of MCH care. The second question is whether existing PFS such as suggestion boxes in the study settings are effective. Literature has suggested that infective PFS may impact both their access and utilization by patients silencing their critical voices in healthcare service improvement [32, 38–40]. Although one patient described offering suggestions, the accounts of many other participants indicate that PFS are ineffective in the study settings. Even in a few healthcare facilities where available, the findings indicate that suggestion boxes, for example, are weakly utilized by patients. Non-utilisation and ineffectiveness of suggestion boxes are also indicated by providers’ description of receiving no suggestions/complaints for a very long time. Consequently, some patients often seek to utilise the media and political forums. Non-utilization is not only rooted in their unavailability in many healthcare facilities but also in poor patients’ awareness of the existence of PFS, uncertainties and fears, structural barriers, and non-responsiveness of PFS. Similar findings are documented by Gurung et al. [40] when examining why patients do not complain in Nepal, with improving patient awareness about the service they deserve and the existence of PFS as well as strengthening feedback mechanisms proposed as solutions. Likewise, similar findings have been reported in a study on patient trust in doctors that was conducted in the same settings between 2015 and 2016 and examined factors shaping trust in providers as one of its objectives [36]. This indicates that the challenges of PFS in the study settings are persistent calling for remedial strategies. A suggestion is therefore made that efforts to promote the effectiveness of PFS need to be implemented alongside activities that seek to create awareness among patients about the value of their engagement in service quality improvement through feedback mechanisms.

The question of who works on the complaints/suggestions dominates patients’ descriptions. Concerns of complaints/suggestions reaching the same people indicate patients’ fear of provider’s retaliation with some proposing the need for an independent agency or entity to handle them in healthcare settings. Looking at previous literature, there have been several discussions regarding how complaints need to be handled in the healthcare sector. Some researchers suggest placing PFS as a key requirement within the laws governing medical practice and empowering medical regulatory bodies, commissions, or committees to take a leading role [41–43]. This may partly remedy the politicization of medicine in some countries, for instance, SSA where such practice is prevalent.

Within Tanzania, patient feedback/complaints handling is among the prescribed duties of the committee on ethics and professional conduct in the proposed Medical and Dental Practitioners Act [16, 44]. However, the challenge to the fulfilment of this duty relates to how patients’ complaints/suggestions reach the committee which often relies on the media and political channels. Furthermore, some researchers recommend handling complaints/suggestions in hospitals where they originate for service improvement noting that an independent procedure is a necessity [29–31, 42]. For instance, in Australia, Taylor et al. [30] indicate that regardless of the outcome at the hospital, the patient complaint/suggestion is always forwarded to an independent government agency to ensure oversight. In Holland, van der Wal and Lens [42] noted some improvement in Hospital care following the implementation of an independent PFS procedure. However, while this may be an option for bigger Hospitals with complex structures and many staff, the question of how the ‘independent’ procedures can be ‘independent’ and who handles such complaints/suggestions within small healthcare facilities with fewer staff in rural settings may limit its applicability. Likewise, the practicality of an independent agency for gathering and handling patients’ complaints and concerns as suggested may need further investigations.

Some countries in SSA appear to have embraced a form of an independent body within the structure of health care facilities. Examples are Tanzania and Kenya which have placed health facility governance committees (HFGC) within the healthcare facility management structures [44, 45]. The HFGC comprising a mix of community members and providers is charged with other duties such as financial planning, management, and resource mobilization on top of promoting health worker performance and promoting smooth relationships between community members (service users, patients) and the health facility (providers) [32, 44, 45]. HFGCs, therefore, form not only an important body for handling complaints at the healthcare facility level but also may act as a PFS by itself. However, poor community awareness of their existence, underrepresentation of the community within the committee and low member motivation has rendered them ineffective [44, 45]. This implies that, before considering PFS an entry point to addressing patient dissatisfactions in patient-doctor relationships, the questions of who handles the complaints need to be addressed and patients may need to be made aware of their existence.

Whilst literature has mainly discussed written complaints/suggestions (patient letters) and verbal complaints [29, 30, 40], the technological advancements call for eyeing of other options which can empower the usage of social media, mobile phones, or anonymous questionnaires. However, the question as to whether these methods can work in low-income rural settings, can guarantee confidentiality and protection of patients against provider retaliation remains central to their effectiveness. This suggests that other context-specific forms of PFS need to be examined more broadly.

## Limitations

This paper offers insights into the potential benefits of PFS in therapeutic relationships in rural Tanzania. However, the concerns related to PFS emerged as a theme during the analysis of drivers of poor nurse-client relationships within the first three transcripts. This resulted in adjusting the interview guide to include probes related to PFS (see description of interview guide in the methods section). While a more extensive and topic-specific study may yield richer information, the findings of this study offer a starting point for such inquiry. Furthermore, the involvement of male patients in the study was limited. The farming season with Males taking a leading role in Sukuma land and a focus on MCH where males are weakly engaged in rural contexts [36] may explain the limited number of male participants. Since gender may influence patient experiences within healthcare settings and the meanings assigned to them [36], future studies including more male participants are recommended.

## Conclusion

Effective PFS may have the potential to improve interpersonal relationships between patients and providers in PHC. When patients complain and suggestions are effectively handled, changes instituted, and feedback is given, the long-term impact may be reduced dissatisfactions, particularly with the interpersonal aspect of care and increased patient trust in doctors, healthcare seeking, engagement in care and continuity. However, the findings indicate that the availability, user-friendliness, and responsiveness of PFS continue to be challenging. A call is made to providers and health administrators to maximise the availability, accessibility, and effectiveness of PFS as it is an important tool for reducing interpersonal tensions in MCH care and improving the quality of healthcare services. Researchers are welcome to investigate strategies for enhancing the effectiveness of PFS as both a useful entry point for strengthening provider client-relationships and, a tool for improving patient service uptake, continuity with care and satisfaction.

### Supplementary Information

**Additional file 1.**

## Data Availability

The dataset(s) supporting the conclusions of this article are included in the article. Additional data on the HCD process that are not part of the published article will be available on request from the AKU through the corresponding author (KI). Some data may not be publicly available for ethical reasons (i.e., information that could compromise the privacy of research participants).
