# Supplementary material for: The perceived benefits and effectiveness of patient feedback systems in strengthening patient-provider relationships in Rural Tanzania
Source: BMC Health Serv Res. 2023 Nov 3;23:1202. doi: 10.1186/s12913-023-10198-z (PMC10623771; doi:10.1186/s12913-023-10198-z)
Supplement: Supplementary file 1 — Additional file 1. [file 12913_2023_10198_MOESM1_ESM.pdf]

AGA KHAN UNIVERSITY
SCHOOL OF NURSING AND MIDWIFERY

ADAPTED INTERVIEW GUIDE

PROJECT TITLE: IMPROVING NURSE-CLIENT RELATIONSHIPS IN MATERNAL AND CHILD HEALTH CARE IN RURAL TANZANIA: A HUMAN-CENTERED DESIGN (HCD) APPROACH

PART 1: FOCUS GROUP DISCUSSION WITH NURSES AND CLIENTS

- *Ensure availability of a quiet and safe venue*
- *Welcome all the participants.*
- *Read the consent form.*

1: PARTICIPANTS DEMOGRAPHIC INFORMATION

PARTICIPANT	AGE	GENDER	MARITAL STATUS	LEVEL OF LICENCE (NURSES ONLY)	HIGHEST LEVEL OF EDUCATION	LEVEL OF HEALTH FACILITY	YEARS OF MCH CARE EXPERIENCE
1							
2							
3							
4							
5							
6							
7							
8							

Facilitator____________________

Notetaker____________________

Starting time_________________

2: FGD QUESTIONS

- What does a good nurse-client relationship mean to you? (*Probe: ever experienced good relationship with nurses/clients? What happened?)*
- What does a bad/poor nurse-client relationship mean to you? (*Probe: ever experienced bad relationship with nurse/clients? What happened?)*
- What are the benefits of having a good relationship with your nurse/client in MCH care?
- What are the consequences of having a bad relationship with your nurse/client in MCH care? *(Probe: consequence to nurses, consequence to clients, consequence to health system?*
- What contributes to a bad relationship between nurses and their clients in MCH care in Shinyanga? *(Probe: Nurse factors, client factors, health system factors?)*
- What are the existing strategies for strengthening nurse-client relationships in MCH care in Shinyanga?
- What are your recommendations (strategies) for strengthening nurse and client relationships within MCH care in Shinyanga? *(Probe: How each strategy mentioned can be made much more successful? Key considerations when implementing each strategy? What are the possible barriers that can impact the successful implementation of each strategy mentioned?)*
- In a couple of previous interviews, Patient Feedback Systems (PFS) emerged as a key strategy for strengthening nurse-client relationships, what do you think about PFS?

    a. What are the potential benefits of PFS?
    b. What are the common PFS available?
    c. What can you say about their accessibility?
    d. How are patients using them?
    e. How are the complaints handled?
    f. How is the feedback provided?

- Any other comment in relation to the nurse-client relationship in Shinyanga?

THANK YOU End Time, _______________________

AGA KHAN UNIVERSITY
SCHOOL OF NURSING AND MIDWIFERY

PART 2: KII WITH MCH ADMINISTRATORS

- *Ensure availability of a quit and safe venue*
- *Welcome the participant.*
- *Read the consent form.*

Interviewer ____________________
Starting time___________________

1: PARTICIPANT DEMOGRAPHIC INFORMATION

1. Participant title: ______________________
2. Age__________________________________
3. Gender_______________________________
4. Highest level of Education____________
5. Marital Status________________________
6. Years of MCH leadership_______________

2: KII QUESTIONS

- What are your roles and responsibilities in relation to MCH care?
- What does a good nurse-client relationship mean to you? (*Probe: ever received client complements of nurses? What happened?)*
- What does a bad/poor nurse-client relationship mean to you? (*Probe: ever handled clients' complaints about nurses? What happened?)*
- What are the benefits of having a good relationship with your nurse/client in MCH care?
- How a bad relationship between nurses and clients have impacted MCH care in Shinyanga? *(Probe: consequence to nurses, consequence to clients, consequence to the health system?*
- What contribute to a bad relationship between nurses and their clients in MCH care in Shinyanga? *(Probe: Nurse factors, client factors, health system factors?)*
- What are the existing strategies employed for strengthening nurse-client relationships in MCH care in Shinyanga?
- What are your recommendations (strategies) for strengthening nurse and client relationships within MCH care in Shinyanga? *(Probe: How each strategy mentioned can be made much more successful? Key considerations when implementing each strategy? What are the possible barriers that can impact the successful implementation of each strategy mentioned?)*
- In a couple of previous interviews, Patient Feedback Systems (PFS) emerged as a key strategy for strengthening nurse-client relationships, what do you think about PFS?

    a. What are the potential benefits of PFS?
    b. What are the common PFS available?
    c. What can you say about their accessibility?
    d. How are patients using them?
    e. How are the complaints handled?
    f. How is the feedback provided?
- Any other comment in relation to the nurse-client relationship in Shinyanga?

THANK YOU

End Time,_______________________
